# ECM Depletion Is Required to Improve the Intratumoral Uptake of Iron Oxide Nanoparticles in Poorly Perfused Hepatocellular Carcinoma

**DOI:** 10.3389/fonc.2022.837234

**Published:** 2022-02-22

**Authors:** Yen Ling Yeow, Jiansha Wu, Xiao Wang, Louise Winteringham, Kirk W. Feindel, Janina E. E. Tirnitz-Parker, Peter J. Leedman, Ruth Ganss, Juliana Hamzah

**Affiliations:** ^1^Harry Perkins Institute of Medical Research, Centre for Medical Research, QEII Medical Centre, The University of Western Australia, Nedlands, WA, Australia; ^2^Centre for Microscopy, Characterisation and Analysis, The University of Western Australia, Nedlands, WA, Australia; ^3^Curtin Medical School and Curtin Health Innovation Research Institute, Curtin University, Bentley, WA, Australia

**Keywords:** extracellular matrix, peptide targeting, hepatocellular carcinoma, nanoparticles, tumor necrosis factor, immune cells, perfusion, magnetic resonance imaging

## Abstract

Improving tumor access for drug delivery is challenging, particularly in poorly perfused tumors. The availability of functional tumor blood vessels for systemic access is vital to allow drugs or imaging agents to accumulate in the tumor parenchyma. We subjected mice engineered to develop hepatocellular carcinoma (HCC), to treatment with tumor necrosis factor alpha (TNFα) conjugated to a CSG peptide (CSGRRSSKC). CSG binds to the laminin-nidogen-1 complex of the extracellular matrix (ECM) in HCC. When produced as a recombinant fusion protein, the TNFα-CSG functions as an ECM depletion agent *via* an immune-mediated mechanism to improve tumor perfusion. Tumor perfusion in HCC was dramatically improved after daily intravenous (i.v.) injection of 5 µg TNFα-CSG for five consecutive days. Following treatment, we assessed the tumor accessibility to accumulate an imaging agent, superparamagnetic iron-oxide nanoparticles (IO-NP). Here, we compared the passive delivery of an i.v. dose of IO-NP in HCC following ECM depletion after TNFα-CSG treatment, to the intratumoral accumulation of a comparable dose of CSG-targeted IO-NP in HCC with intact ECM. Magnetic resonance imaging (MRI) T_2_-weighted scans and T_2_ relaxation times indicate that when the tumor ECM is intact, HCC was resistant to the intratumoral uptake of IO-NP, even when the particles were tagged with CSG peptide. In contrast, pre-treatment with TNFα-CSG resulted in the highest IO-NP accumulation in tumors. These findings suggest poorly perfused HCC may be resistant to molecular-targeted imaging agents including CSG-IO-NP. We demonstrate that specific ECM depletion using TNFα-CSG improves nanoparticle delivery into poorly perfused tumors such as HCC.

## Introduction

Systemic delivery of therapeutics and imaging agents in tumors relies primarily on accessibility *via* tumor blood vessels (1). The passive delivery of non-targeted agents in tumors is based on the enhanced permeability and retention (EPR) effect of leaky tumor vasculature, resulting in higher accumulation in tumors than in normal tissues (2). However, this passive delivery *via* the EPR effect is insufficient to allow macromolecules and nanoparticles to extravasate and accumulate in the parenchyma of solid tumors (3). More recently, ligands with specific binding affinity to unique molecular tumor targets have been developed for the delivery of therapeutic and imaging payloads including nanoparticle-based contrast agents (4). We have previously reported a tumor extracellular matrix (ECM)-targeting peptide, CSG, that specifically binds to the aberrant network of laminin-nidogen-1 complex in a number of mouse and human solid tumors including breast, pancreatic and liver cancers (5). When CSG is linked to superparamagnetic iron-oxide nanoparticles (IO-NP), CSG-IO-NP accumulate selectively in the stromal ECM, for instance in pancreatic neuroendocrine tumors (PNET) (6).

However, intratumoral binding to specific molecular targets relies on a functional circulation *via* tumor blood vessels. This remains a challenge for clinical and experimental tumors that are heterogeneously perfused and thus represents a significant barrier to drug delivery (7–11). In particular, tumors that develop in the liver, including hepatocellular carcinoma (HCC) are difficult to target for nanoparticle delivery (8–13). Whilst the normal liver is very well-perfused through hepatic arterial and portal venous blood flows, HCC nodules are fed primarily *via* arterial blood supply (8, 13–15). Hence, intravenously injected nanoparticles accumulate preferentially in the normal liver (10, 13). Furthermore, the heterogeneity of blood perfusion in HCC can impede intratumoral uptake of nanoparticles. Dysplastic nodules and early-stage HCC are hyper-vascularized and well-perfused but late-stage and poorly differentiated HCC showed significant decrease in arterial blood supply. Injection of imaging materials detectable by contrast-enhanced computed tomography, ultrasonography and magnetic resonance imaging (MRI) revealed a perfusion defect in advanced-staged HCC compared to the non-cancerous regions (8, 10, 11). Therefore, the use of nanoparticle-based imaging contrast enhancement in HCC diagnosis is often based on non-specific accumulation in the hepatic tissues and small peritumoral vessels surrounding the tumors (16, 17).

We have previously shown that CSG-IO-NP targets the stromal ECM in a PNET tumor model and is a contrast agent superior to untargeted IO-NP in detecting tumor stroma by MRI (6). In addition, we have developed a therapeutic approach to improve tumor perfusion in PNET and breast cancer models using an immune modulating cytokine tagged to CSG peptide, TNFα-CSG. TNFα-CSG treatment induces immune-mediated ECM depletion, which alleviates the compression on the tumor blood vessels and enhances tumor perfusion. TNFα-CSG-treated tumors are then more accessible for systemic uptake of nanoparticles (5).

In this study, we assessed the accumulation of CSG-IO-NP and untargeted IO-NP in poorly perfused HCC with intact ECM, and the passive uptake of IO-NP following ECM-depletion by TNFα-CSG treatment. Our findings indicate that IO-NP have poor access to HCC, even when the particles were targeted with CSG peptide. However, IO-NP accumulation in HCC is significantly improved following pre-treatment with TNFα-CSG. Thus, we demonstrate that TNFα-CSG is a useful ECM-depletion agent that can significantly improve HCC perfusion and facilitate the passive delivery of IO-NP.

## Methods

### Animal Models

ALB-Tag mice (on a C3H background) express the oncogene SV40 Large T antigen (Tag) under the control of the mouse albumin gene promoter (ALB), and develop spontaneous HCC as previously described (18). RIP1-Tag5 mice express Tag under the control of the rat insulin gene promoter (RIP) in pancreatic β cells, and develop spontaneous PNET as previously described (19). ALB-Tag or RIP1-Tag5 mice with advanced tumor nodules were used at ≈10–12 or ≈29–30 weeks of age, respectively. All mice were kept under pathogen-free and temperature-controlled conditions in individually ventilated cages, exposed to a 12-hour day-to-night cycle. A minimum of n = 3 mice per group were used in each study. All animal experiments were performed in accordance with the Australian code for the care and use of animals for scientific purposes at the University of Western Australia (UWA) Animal Facility with approval from the UWA Animal Ethics Committee.

### *In Vivo* Lectin Perfusion

Mice were intravenously (i.v., tail vein) injected with 1 μg/μL of tomato lectin (Lycopersicon esculentum conjugated to either FITC or DyLight^®^ 594; Vector). After 10 min of circulation, the mice were heart-perfused with 2% neutral-buffered formalin (w/v) and tumors with surrounding normal tissue were frozen in the O.C.T. compound (Tissue-Tek^®^).

### CSG Peptide and TNFα-CSG Synthesis

Synthetic carboxyfluorescein (FAM)-labelled CSG (molecular weight=1,612.17) was kindly provided by Dr. Vankata Ramana Kotamraju (Sanford Burnham Prebys Medical Discovery Institute, La Jolla, CA, USA). Recombinant murine TNFα-CSG (18.9 kDa) was produced as described previously (5, 20).

### *In Vivo* Peptide and TNFα-CSG Binding

Tumor-bearing mice were injected i.v. with 100 µL of either 1 mM FAM-CSG, FITC-TNFα-CSG or FITC-TNFα in phosphate buffered saline (PBS). After 1 h, animals were euthanised and subjected to transcardial perfusion with sterile PBS to remove unbound peptides or proteins. Tissues, including tumor, heart, liver, spleen, kidney, vertebrae, lung and pancreas were excised and imaged under a UV illuminator (Illumatool, Lightools Research, CA, USA) for assessment of green fluorescence intensity. Tissues were embedded in O.C.T. (Tissue-Tek^®^) and stored at -80°C for histology analysis.

### TNFα-CSG Treatment

Tumor-bearing mice were i.v. injected once per day with PBS, CSG (0.8 µg), or TNFα-CSG (5 µg) for 4 to 5 consecutive doses. Tumors were analyzed for immune cell infiltration, lectin perfusion and IO-NP uptake within 48 h after final treatment, unless stated otherwise.

### Analysis of Intratumoral Nanoparticle Uptake by Magnetic Resonance Imaging

Superparamagnetic iron oxide nanoparticles (IO-NP) with and without CSG conjugation were prepared and analyzed as previously published (6, 21–23). Untargeted IO-NP (6) were injected i.v. (100 µL, 5 mg/kg Fe) in tumor-bearing mice within 24 h after final treatment with TNFα-CSG or CSG peptide. In parallel, untreated tumor-bearing mice received an i.v. dose of CSG-IO-NP as described previously (6). After 4 h circulation, mice were perfused with sterile PBS. Tissues were collected, fixed in 2% formalin for 2 h and embedded in 2% agarose. *Ex vivo* MRI scans were performed at 9.4 T with a Bruker BioSpec 94/30 magnet, Avance III HD console and ParaVision 6.0.1 acquisition software as described previously (5, 6). T2* and T2 parameter maps were calculated from the MGE and MSME datasets, respectively, using the ParaVision 6.0.1 macro *fitinlsa*, which fits the signal for each pixel according to a mono-exponential decay. Image analysis was performed using ImageJ. Tumor volume and IO-NP-induced changes in MRI signal level were measured by using the *Image Display and Processing Tool*. Regions of interest (ROI) for each tumor was manually defined using the *track* tool, based on the MSME image with TE = 8 ms. Statistical analyses for tumor volumes were obtained by combining the ROI statistics on an image slice by slice.

### Flow Cytometry Analysis

Tumors and normal liver tissues were excised from PBS-control and TNFα-CSG-treated C3H and ALB-Tag mice, minced and incubated in Dulbecco’s Modified Eagle Medium (DMEM) high-glucose medium containing 0.1 mg/ml DNase I (Sigma) and 0.5 mg/ml collagenase IV (Sigma) for 1 h at 37°C under gentle rotation. The cell suspension was passed through a 70-μm membrane filter and subsequently washed with FACS buffer [1% BSA (w/v, Sigma) in PBS]. For analysis of immune cells, viable cells were stained with the following antibodies: CD11b-A488 (M1/70, BD Pharmingen, 1:100), CD68-A647 (FA-11, Biolegend, 1:100), CD8-FITC (53-6.7, eBioscience, 1:100), CD4-FITC (GK1.5, eBioscience, 1:100) and CD45-A700 (30-F11, eBioscience, 1:100). Cells were imaged on a BD SORP Fortessa and analyzed on BD FACSDiva software version 8.0.1 (BD Biosciences, USA).

### Histology Analysis

Tissue distribution of bound lectin, FAM-CSG and FITC-TNFα-CSG was detected on 8 μm tissue sections based on their fluorescence intensity. The intensity of FITC-TNFα-CSG and FITC-TNFα in tissues were amplified with anti-fluorescein antibody (polyclonal, LifeTechnologies). For co-staining analysis, the following antibodies were used: anti-SV40 Large T antigen antibody (polyclonal, Santa Cruz, 1:100) anti-CD31 (390; ebioscience, 1:100), anti-laminin (polyclonal; Millipore, 1:200) and anti-nidogen-1 (polyclonal; Millipore, 1:100), CD11b (M1/70, BD Pharmingen), CD68 (FA-11, Abcam), CD8 (53-6.7, eBioscience, 1:100) and CD4 (GK1.5, eBioscience, 1:100). For secondary detection, fluorescence-labelled 488-, 594- or 647-conjugated anti-rat, rabbit or goat IgG (LifeTechnologies) were used. Images were captured on a Nikon Ti-E microscope (Nikon Instrument Inc., NY, USA) or 3DHISTECH Slide Scanner (3DHISTECH, Budapest, Hungary). Image analysis and quantification were performed using NIS software modules (version 4.0).

### Statistical Analysis

Statistical analyses were performed using GraphPad Prism 7 (GraphPad Prism Software, San Diego, CA, USA). Data were analyzed by the Student’s t-test (two-tailed) or one-way analysis of variance (ANOVA) as indicated. A p value < 0.05 was considered statistically significant. Error bars indicate standard error of the mean (SEM). Experiments were carried out in an unblinded fashion.

## Results

### Accessibility of ALB-Tag HCC to Lectin, CSG Peptide and TNFα-CSG

**Figure 1:**
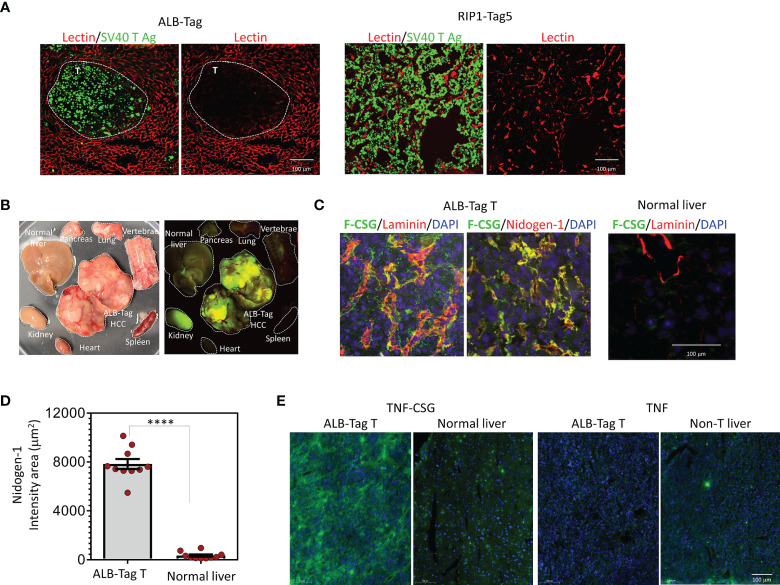
HCC accessibility to lectin and binding specificity to CSG peptide and TNFα-CSG. **(A)** Lycopersicon Esculentum (Tomato) Lectin DyLight^®^ 594 was injected i.v. in mice bearing ALB-Tag HCC or RIP1-Tag5 tumors. Representative micrographs on indicated tissues show lectin-painted vessels (red) in tumors (T) stained with anti-SV40 Large T antigen antibody (green). **(B)** Mice bearing ALB-Tag HCC were i.v. injected with 0.1 mmol of FAM-CSG, and tissues were collected after 1 h of circulation. Photographic image of collected tissues under bright light (left) and UV illuminator (right) are shown. Peptide binding is shown in tumors (green) but not in normal tissues. The kidney is the clearance organ. **(C)** HCC tumors from ALB-Tag mice and normal liver from C3H mice were co-stained for laminin and nidogen-1. Representative micrographs show FAM-CSG binding (green) and laminin or nidogen-1 expression (red). Co-localisation is indicated in yellow. Scale bar 100 µm. **(D)** Quantitative analysis of nidogen-1 staining per field of each tumor or normal liver, as shown in panel **(C)** and mean ± SEM (****P < 0.0001 by Student’s *t*-test). **(E)** Analysis of FITC-TNFα-CSG and FITC-TNFα binding *in vivo* detected with anti-FITC antibody (green) and nuclear staining (DAPI) in indicated tissues. Scale bars: 25 μm.

Intratumoral perfusion requires functional blood vessels. We first evaluated blood vessel functionality in ALB-Tag HCC in comparison with PNET in RIP1-Tag5 mice using i.v.-injected lectin binding to blood vessels as a surrogate marker for tumor perfusion. **Figure 1A** compares the ALB-Tag and RIP1-Tag5 tumors that were positive for SV40 T antigen. Whilst all vessels in the surrounding normal liver were positive for lectin, ALB-Tag HCC showed only weak traces of lectin-painted blood vessels. In contrast, RIP1-Tag5 tumors visibly displayed lectin-painted blood vessels. This finding suggests that the ALB-Tag HCC is more difficult to access systemically than RIP1-Tag5 tumors.

However, as shown in **Figure 1B**, i.v.-injected fluorescein-conjugated CSG peptide (FAM-CSG), homed specifically into HCC but not other normal tissues including liver. In HCC, FAM-CSG colocalized with its target receptor, the laminin and nidogen-1 complex, which is expressed in tumor ECM (**Figure 1C**). As indicated by nidogen-1 staining in **Figure 1D**, HCC expressed CSG receptor at levels 25-fold higher than the normal liver. In addition, the i.v.-injected FITC-labeled TNFα-CSG also accumulated strongly in HCC compared to the liver tissue (**Figure 1E**). The unconjugated TNFα showed limited tumor uptake (**Figure 1E**). These data demonstrate that CSG-tagged molecules accumulate in HCC tumor parenchyma, and when conjugated to TNFα, have the potential to influence the ECM perfusion barrier.

### Immune-Mediated Effects of TNFα-CSG in Improving HCC Perfusion

**Figure 2:**
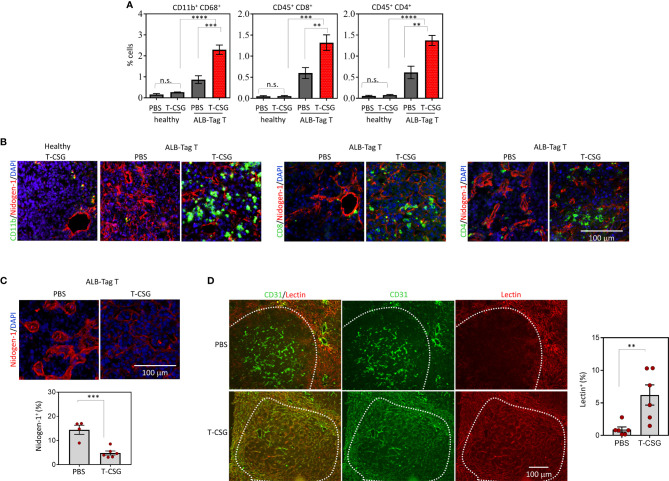
Intratumoral effects of TNFα-CSG on immune cell infiltration, ECM content and lectin perfusion. **(A)** Quantification of immune cells in HCC and normal liver in ALB-Tag and C3H mice treated with daily i.v. injection of PBS or 5 µg TNFα-CSG for five consecutive days. Bar charts show mean ± SEM of cell counts for macrophages (CD11b^+^/CD68^+^) and T cells (CD45^+^ CD8^+^ or CD45^+^ CD4^+^) in each treatment group, **P<0.01, ***P < 0.001, ****P< 0.0001 and n.s. (not significant) when P > 0.05 (by one-way ANOVA test). **(B)** Co-staining of HCC and normal liver sections treated with five daily doses of indicated compounds, as in panel **(A)**. Micrographs depict CD11b^+^ cells and CD8^+^ and CD4^+^ T-cell (green) infiltration particularly around nidogen-1(red)-positive structures. Scale bar: 100 μm. **(C)** Top: Representative tumors from indicated treatment groups indicating changes to nidogen-1 content (red). Bottom: Quantitative analysis of nidogen-1 staining/field/tumor in PBS and TNFα-CSG treatment group and mean ± SEM (***P < 0.001 by Student's *t*-test). **(D)** Co-staining of lectin-perfused HCC [following treatment as in panel **(B, C)**] with CD31 (red, blood vessels). Micrographs show co-localisation (yellow) of lectin and CD31 staining in tumors. Scale bar: 100 μm. Right: Data show the fraction of lectin-positive staining in individual tumors and mean ± SEM (**P < 0.01 by Student’s *t*-test).

We have previously demonstrated that directing TNFα-CSG to tumor ECM triggers immune cell infiltration which in turn delivers a cocktail of ECM-degrading proteases leading to specific tumor ECM breakdown (5). To assess the intratumoral effect of TNFα-CSG in HCC, ALB-Tag mice and age-matched wild-type C3H mice were treated with an i.v. dose of 5 μg TNFα-CSG per day for five consecutive days. Tumors from ALB-Tag mice and normal liver from C3H mice were analyzed two days after the final treatment for immune infiltrates, ECM content and lectin binding (**Figure 2**). ALB-Tag HCC tumors treated with TNFα-CSG showed significantly higher infiltration of macrophages (CD68^+^/CD11b^+^), CD8^+^ and CD4^+^ T cells compared to PBS-treated tumors (gating strategy and FACS plots are shown in **Supplementary Figure S1**). The TNFα-CSG-induced immune cell infiltration was specific to tumors as the immune cell status in normal liver tissue remained unaffected by the TNFα-CSG treatment (**Figures 2A, B**). Intratumorally, the immune infiltrates accumulated mainly in ECM areas positive for nidogen-1 expression (**Figure 2B**).

Next, we assessed changes to the tumor ECM content in response to TNFα-CSG treatment. Data in **Figure 2C** highlight that nidogen-1 staining in TNFα-CSG-treated tumors was significantly lower than in the control PBS-treated tumors. This ECM-depletion effect is consistent with our previous data in other tumor models (5). To determine the effect of ECM reduction on tumor perfusion, lectin binding to tumor blood vessels was compared in TNFα-CSG- treated versus control tumors. **Figure 2D** shows representative tumors with lectin-painted blood vessels co-stained for the blood vessel marker CD31. The indicated micrographs show a lack of lectin-staining in HCC with intact ECM, even though the tumors were positive for vessel staining (CD31). TNFα-CSG-treated tumors show visible lectin-painted blood vessels (lectin^+^/CD31^+^). The quantitative analysis showed a 6-fold increase in lectin-painted tumor blood vessels in TNFα-CSG-treated HCC (**Figure 2D**). These data demonstrate that TNFα-CSG improves blood vessel function in HCC.

### Accessibility of IO-NP in Intact and ECM-Depleted HCC

**Figure 3:**
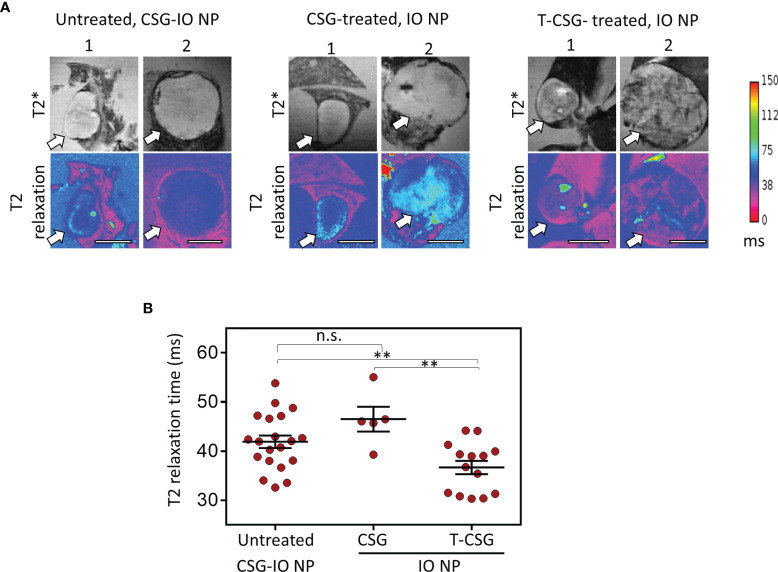
Accumulation of CSG-IO-NP and IO-NP in HCC with intact and/or reduced ECM detected by MRI. **(A)** Representative images of T2*-weighted (top) and T2 relaxation maps (magenta, bottom) from *ex vivo* MRI scans of liver with HCC (tumor sizes, <5 mm and >5 mm in diameter, indicated by arrow). Scale bars: 4 mm. Left: HCC with intact ECM after 4 h *in vivo* circulation of CSG-IO-NP. Middle: HCC treated with 5 × 0.8 µg CSG peptide and then i.v.-injected with untargeted IO-NP. Right: HCC treated with 5 × 5 µg TNFα-CSG and then i.v.-injected with untargeted IO-NP. **(B)** Reduction in T2 relaxation time indicates the relative increase of IO-NP in individual tumors and mean ± SEM per group (**P < 0.01 and n.s. when P > 0.05 by one-way ANOVA test).

Next, we assessed the intratumoral uptake of CSG-IO-NP in HCC by performing *ex vivo* MRI scans of the ALB-Tag HCC tumors following 4 h *in vivo* circulation of CSG-IO-NP. In parallel, we include the MRI scans of ALB-Tag HCC injected with untargeted IO-NPs, following TNFα-CSG or CSG-peptide control treatment at doses and dosing frequencies as described in **Figure 2**. **Figure 3A** illustrates T2* and T2 relaxation images of scanned HCC based on their small (diameter < 5 mm) and large (diameter > 5 mm) sizes. Based on changes in the T2 relaxation time which indicate the relative amount of IO-NP in tumors, our data (**Figure 3B**) demonstrate that CSG-IO-NP did not effectively accumulate in HCC, unlike previously reported in the RIP1-Tag5 tumors (6). There was no significant difference between CSG-IO-NP and the untargeted IO-NP in HCC with intact ECM. However, our data showed that TNFα-CSG pretreatment induced significantly higher IO-NP accumulation in HCC, when compared to control tumors treated with either CSG-targeted or untargeted IO-NP.

**Figure 4:**
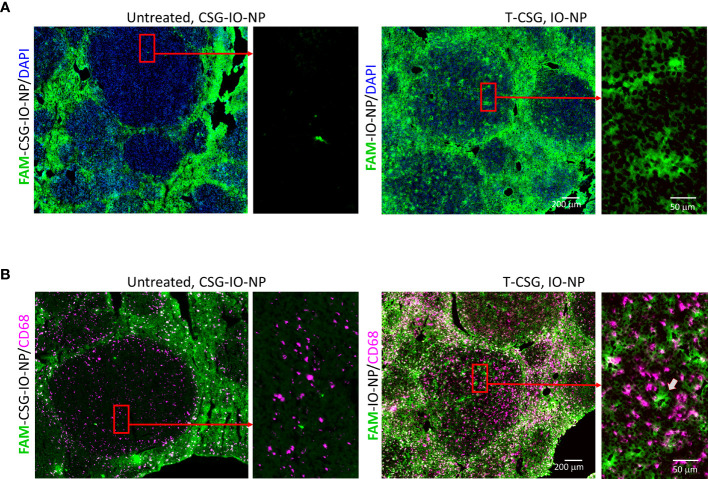
Intratumoral localisation of IO-NP. Liver tissue sections from indicated treatment group 4 h after an i.v. injection of FAM-labelled CSG-IO-NP and IO-NP (as described in **Figure 3**) were analyzed for IO-NP distribution. **(A)** Representative micrographs show detection of FAM-labelled CSG-IO-NP and IO-NP, which were amplified by immunostaining with anti-FITC antibody (green). Rectangular box: Selected regions in HCC were compared for IO-NP accumulation at higher magnification. **(B)** Corresponding tissues in **(A)** were co-stained for CD68^+^ cells (infiltrating macrophages, red). Arrow: An area within tumor positive for IO-NP that lacks CD68^+^ cells. Scale bars: 200 and 50 μm.

We further confirmed the distribution of IO-NP in HCC by immunofluorescence analysis. **Figure 4** shows micrographs of HCC with and without ECM depletion, immunologically stained for traces of FAM-labelled CSG-IO-NP and FAM-IO-NP and distribution of infiltrating macrophages (CD68^+^). The micrographs depict higher traces of IO-NP in TNα-CSG-treated HCC compared to CSG-IO-NP in the HCC with intact ECM (**Figure 4A**). The IO-NP distribution in response to TNFα-CSG treatment exceeds the number of CD68^+^ macrophages (**Figure 4B**), suggesting that intratumoral accumulation of IO-NP was independent of phagocytic uptake of the nanoparticles by the infiltrating macrophages. Our findings suggests that pre-treatment with perfusion-promoting agents, such as TNFα-CSG, may improve the delivery of nanoparticles especially for poorly perfused tumors, such as ALB-Tag HCC.

## Discussion

Our study demonstrates that whilst EPR may be sufficient for some small drug molecules to penetrate solid tumors, poorly perfused tumors may remain inaccessible for larger molecules, including nanoparticles such as IO-NP. Even when IO-NP were tagged with CSG peptide, which is known to improve intratumoral uptake through CSG-specific binding to the aberrantly expressed tumor ECM (6), poorly perfused ALB-Tag HCC remained resistant to IO-NP uptake. IO-NP particles mainly accumulated in the normal liver. However, a short treatment with TNFα-CSG to increase tumor perfusion resulted in significant improvement in the accumulation of IO-NP in the otherwise impenetrable HCC. Therefore, in addition to the normal clearance activity of the liver, these findings highlight that inadequate perfusion of the tumor represents a major barrier that may significantly affect drug delivery and treatment efficacy in HCC.

Systemically injected agents, including nanoparticles, circulate in the blood until they are cleared from the circulation and eliminated from the body either by renal clearance, or by the liver *via* hepatobiliary elimination (15). The liver clearance pathway makes it particularly challenging to develop strategies for the delivery of agents for liver-associated diseases such as HCC. Hence, the drug delivery approach for HCC needs to take into consideration both passive and active mechanisms to improve intratumoral delivery and to minimize non-specific uptake and rapid elimination by the liver (14). Here, we found that HCC tissues are accessible to small molecules including CSG (<1.5 kDa) and TNFα-CSG (18.9 kDa) but less accessible to lectin conjugates (>70 kDa) and larger molecules such as IO-NP.

Our data indicate that CSG, as a free peptide, effectively escapes the rapid and non-specific liver uptake. CSG is an excellent HCC-targeting agent as it binds specifically to aberrant tumor ECM and not to the normal ECM. However, the use of CSG as HCC-targeting agent for intratumoral delivery is greatly influenced by the type and size of payloads. HCC tissues are inaccessible to the CSG targeted IO-NP, an agent that we have previously shown to be highly effective for intratumoral delivery in tumors with high ECM content such as PNET (6). Similar to the untargeted IO-NP, the CSG-IO-NP mostly accumulated in the non-malignant liver. This finding suggests that the rate of hepatic nanoparticle uptake is quicker and more efficient than CSG-IO-NP access and accumulation in tumors. Our data are consistent with previous reports that found non-specific IO-NP accumulation to be a major challenge for nanomedicines, irrespective of the physical and chemical properties of nanoparticles such as careful control over their surface coating to improve blood half-life, and incorporation of active targeting moieties with specific ligands for enhanced tumor binding (12).

Furthermore, like other treatment-resistant cancers, HCC is comprised of dense stroma with excessive production of ECM (24, 25). Since HCC develops in the setting of cirrhosis in the vast majority of cases, the excessive fibrotic changes with significant matrix deposition and disruption of the hepatic architecture make it extremely challenging for therapeutic agents to penetrate and reach the target cancer cells (26, 27). Our ALB-Tag tumors display some of these microenvironmental features, including the high ECM content and poor perfusion, despite the high level of vascularization of ALB-Tag HCC. TNFα-CSG treatment of mice bearing ALB-Tag HCC increased intratumoral immune cell infiltrates, reduced ECM content and improved tumor perfusion. The observed intratumoral effects in response to TNFα-CSG are consistent with our previous findings in other solid tumor models (5). The ECM-depletion in these tumors was triggered by the protease secretion through increased immune cell infiltration (5). Similar protease-mediated ECM breakdown is likely to occur in HCC, as the TNFα-CSG-treated ALB-Tag tumors showed reduced ECM content. Our ECM-depletion approach to improve tumor perfusion using TNFα-CSG is also consistent with other ECM-reducing strategies to enhance perfusion and increase access to solid tumors (28–31).

An important application of TNFα-CSG treatment may be to sensitize HCC for improved systemic access of nanoparticles such as IO-NP and potentially other HCC-specific therapeutics. CSG binding is conserved in human HCC (5), and thus in addition to its use to enhance diagnostic imaging, TNFα-CSG has the therapeutic potential to improve patient outcome. Currently, most patients are diagnosed with HCC at an advanced stage, when systemic therapy is the only treatment option. The commonly used systemic therapies are the tyrosine kinase inhibitors, sorafenib and lenvatinib, both of which have significant associated toxicity and provide only a marginal survival benefit (32, 33). We have shown that CSG targeting of TNFα dramatically rescued the systemic toxicity associated with untargeted TNFα (5), and therefore ECM-depletion by TNFα-CSG is viable and safe in a preclinical setting. Our data suggest that TNFα-CSG may provide a much-needed novel approach to improve treatment efficacy by increasing systemic access to these drugs.

## Data Availability Statement

The original contributions presented in the study are included in the article/**Supplementary Material**. Further inquiries can be directed to the corresponding author.

## Ethics Statement

The animal study was reviewed and approved by the University of Western Australia Animal Ethics Committee.

## Author Contributions

Conceptualization, JH and RG. Investigation, YY, JW, XW, and KF. Funding acquisition, JH, RG, and PL. Supervision, JH, KF, and LW. Writing, JH. Review and editing, JH, RG, LW, PL, and JT-P. All authors have read and agreed to the published version of the manuscript.

## Funding

This research was supported by Cancer Council WA project grant, Perkins Ride to Conquer Cancer, Liver Cancer Collaboration, NHMRC project grants (APP1058073, APP1121131, APP1157240) to JH and RG, and a Cancer Research Institute Clinic and Laboratory Integration Program (CLIP) Grant to RG. JH and RG are the recipients of the Cancer Council Western Australia Research Fellowship and a Woodside Energy Cancer Research Fellowship, respectively.

## Conflict of Interest

The authors declare that the research was conducted in the absence of any commercial or financial relationships that could be construed as a potential conflict of interest.

## Publisher’s Note

All claims expressed in this article are solely those of the authors and do not necessarily represent those of their affiliated organizations, or those of the publisher, the editors and the reviewers. Any product that may be evaluated in this article, or claim that may be made by its manufacturer, is not guaranteed or endorsed by the publisher.

## Supplementary Material

Supplementary Figure 1 Gating strategies and flow cytometry plots of quantification of CD11b^+^/CD68^+^, CD8^+^/CD45^+^ and CD4^+^/CD45^+^ in normal liver (from C3H mice) and ALB-Tag HCC tumors following i.v. injections of PBS or TNFα-CSG (5 μg dose/day) for 5 consecutive days.
