# Supplementary figures and images for: ECM Depletion Is Required to Improve the Intratumoral Uptake of Iron Oxide Nanoparticles in Poorly Perfused Hepatocellular Carcinoma
Source: Front Oncol. 2022 Feb 22;12:837234. doi: 10.3389/fonc.2022.837234 (PMC8902243; doi:10.3389/fonc.2022.837234)

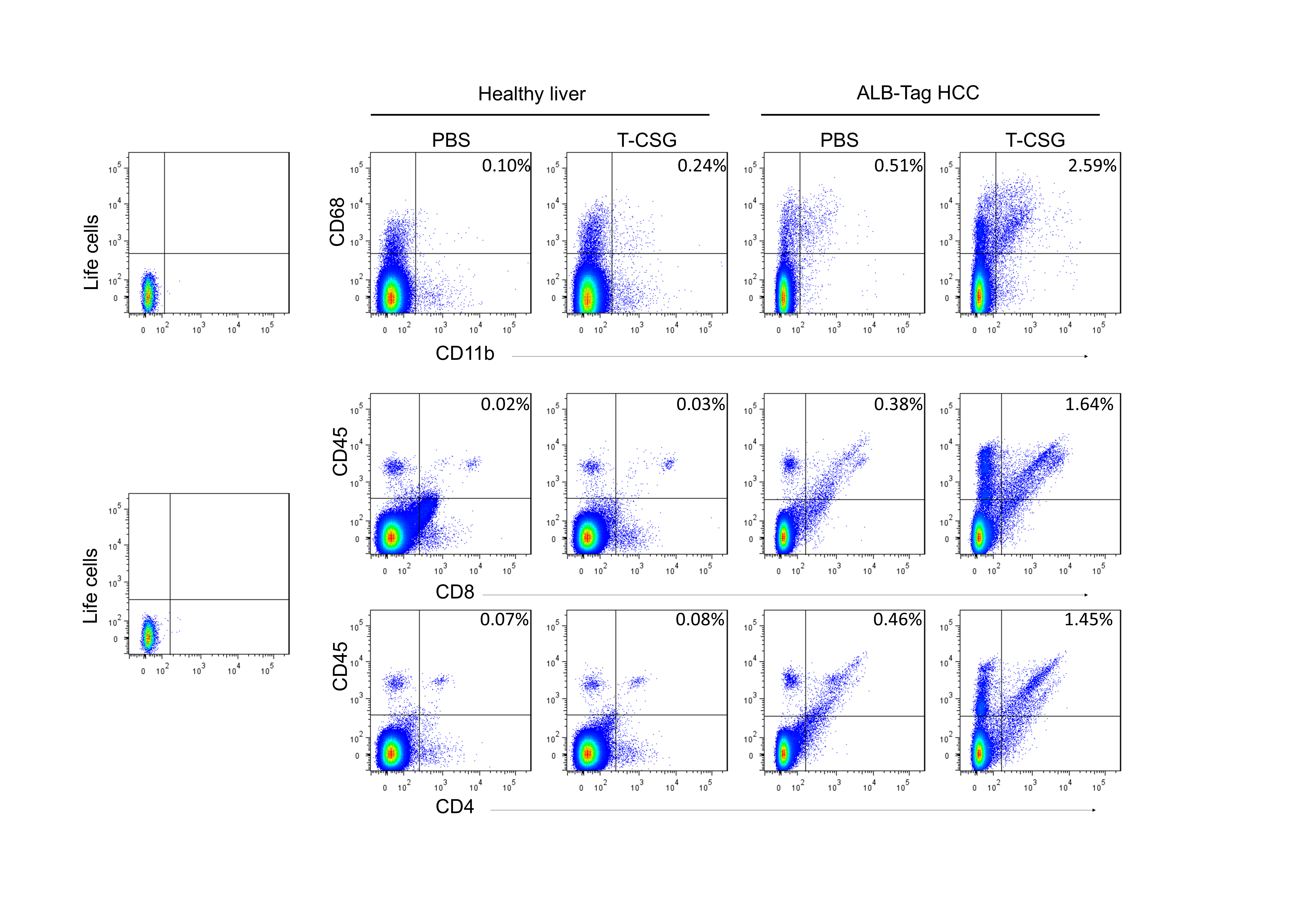

Supplement: Supplementary Figure 1 — Gating strategies and flow cytometry plots of quantification of CD11b+/CD68+, CD8+/CD45+ and CD4+/CD45+ in normal liver (from C3H mice) and ALB-Tag HCC tumors following i.v. injections of PBS or TNFα-CSG (5 μg dose/day) for 5 consecutive days. [file Image_1.tif]
